# Evaluation of X-Chromosome Inactivation Patterns in Patients with Acute Myeloid Leukemia during Remission

**DOI:** 10.5402/2012/971493

**Published:** 2012-10-23

**Authors:** Yousef Mortazavi, Saeid Kaviani, Fatemeh Mirzamohammadi, Kamran Alimoghaddam, Ali Akbar Pourfathollah, Oveis Salehi

**Affiliations:** ^1^Department of Molecular Medicine, Faculty of Medicine, Zanjan University of Medical Sciences, Zanjan 4513956111, Iran; ^2^Department of Hematology, Tarbiat Modares University, Tehran, Iran; ^3^Hematology-Oncology and Bone Marrow Stem Cell Transplantation Research Center, Shariati Hospital, Tehran, Iran; ^4^Department of Immunology, Tarbiat Modares University, Tehran, Iran; ^5^Hormozgan University of Medical Sciences, Hormozgn, Iran

## Abstract

The aim of this study was to evaluate the patterns of X-chromosome inactivation during the remission in acute myeloid leukemia (AML) at the RNA level. Two hundred normal females and 45 female patients with AML entered the study. The frequency of heterozygosity was 48.5% (119/245) for P55, 40% (93/245) for IDS, and only 28.9% (71/245) for G6PD. Some individuals were heterozygous for more than one gene polymorphism. Overall, one hundred normal individuals proved showed to be heterozygous for at least one of the above polymorphisms. 92/100 (92%) normal females showed a polyclonal pattern. Clonal patterns were observed in 44/45 (98%) AML patients at presentation. Of 27 patients who were followed after remission, 23 (85.2%) patients showed a clonal pattern. Ten patients were available for a longer followup (up to 12 months) and the clonal pattern was observed in seven patients. It can be concluded that clonality at remission is a frequent event in AML and does not necessarily mean relapse of the disease. There is also a possibility of conversion of clonality to polyclonality over time.

## 1. Introduction

In contrast with the Y-chromosome, the X-chromosome has a large number of genes that are essential for cell activity. However, a female cell has double the number of X-chromosomes in comparison with a male; male and female cells have approximately an equal amount of X-chromosome-encoded enzymes. X-chromosome inactivation is a process that occurs in all mammals, leading to selective inactivation of alleles on one of the two X-chromosomes in females [1].

Inactivation of the major part of one X-chromosome occurs in the somatic cells of females during early embryogenesis. Therefore, women heterozygous for a polymorphic X- linked gene have a mixture of cells. Some of them expressing one allele others expressing another in their normal tissues [2].

A neoplastic cell population is the progeny of a single cell with a genetic insult, by virtue of fixed inactivation, all the cells contain the same (either the paternal or the maternal) X-chromosome in the active or inactive state. Thus, if only one X-chromosome can be shown to be active in all the cells of a tissue sample, this is evidence of clonality.

Clonality is helpful in differentiation and investigation of pathophysiology of hematologic disorders [3]. Monoclonality usually implies malignant or premalignant disease, so provides strong supportive evidence for the somatic mutation theories of carcinogenesis. 

X-chromosome inactivation has been used widely in female individuals to differentiate reactive hyperplasia from the true clonal cell expansion (neoplasia) arising from somatic mutation. X-inactivation analysis can be performed in the absence of any tumor specific markers, which can be useful in any neoplasm. The clonality of a cell population in hematologic disorders can be determined by a number of different approaches including the use of specific clonal markers such as karyotype abnormalities, gene rearrangement, and point mutations. Each of these methods has some limitations [4]. For instance, the karyotype maybe normal in some neoplastic cells, and a chromosome abnormality is informative only if it is present in a majority of metaphases, and antigen receptor gene rearrangement is helpful only in the lymphoid lineage in lymphoproliferative malignancies such as lymphomas. In the absence of specific clonal markers, other methods can be helpful to determine clonality in female patients, including the use of X-linked G6PD protein polymorphisms, variable number of tandems repeats (VNTRs) and short tondems repeats (STRs). All these methods take advantage of random X-chromosome inactivation during embryogenesis. Each of these techniques has some advantages and disadvantages or limitations [5].

However, DNA methylation does not always perfectly correlates with X-chromosome inactivation; in the most clonality assays, DNA methylation has been used to indicate X-inactivation [6, 7]. The problem appears particularly for some probes such as M27*β*, which would complicate studies of X-chromosome inactivation [8]. In addition it has been reported that leukemic cells may be hypermethylated, which can confuse clonality analysis [9–12]. Unlike DNA sequence polymorphisms, RNA gene polymorphisms can directly measure the gene activity.

AML is the result of clonal transformation of hematopoetic precursors through the acquisition of chromosomal rearrangements. AML comprises 90% of all acute leukemias in adults. The annual incidence is approximately 3.5 per 100,000 and increases with age [13].

Relapses are frequent events after aggressive chemotherapy and allogeneic stem cell transplantation. Laboratory data suggest that AML originates from a rare population of cells, termed leukemic stem cells (LSCs), which are capable of self-renewal, proliferation, and differentiation. These cells may persist after treatment and are probably responsible for the disease relapse [14].

In order to study X-chromosome inactivation in acute myeloid leukemia, here we describe a qauntitative nonradioactive method, analysing the polymorphisms of P55, IDS, and G6PD genes by RT-PCR and the generation of an allele specific restriction site in 200 Iranian healthy females and 45 female patients with AML aged 20–45 years before the treatment and during remission.

## 2. Materials and Methods

### 2.1. Subjects

We have recruited 45 patients with AML who referred to Tehran Shariati Hospital and 200 healthy females without a family history of genetic or neoplastic disorders for X-chromosome inactivation patterns assessment. All patients were diagnosed as AML, based on the clinical symptoms and the lab findings according to FAB classification and molecular genetic analysis. Written inform consent was taken from each patient and the study was approved by the University Ethical Committee. 

### 2.2. Cell Separation

 Peripheral blood mononuclear cells (MNCs) were separated using Ficoll-Hypaque density gradient centrifugation. Granulocytes (PMN) were separated from red cells using 5% Dextran and T cells were separated by rosette formation using sheep RBCs.

### 2.3. DNA Extraction

Isolated cell populations were lysed in 10 mL 1X TNE (Tris 1 M pH 8, NaCl 1 M, EDTA0.5 M pH 8), 2% SDS and proteinase K at 37°C overnight. Then we extracted and purified with the routine procedures.

### 2.4. DNA Amplification

In each amplification, the reaction was made up of 50 *μ*L containing 1X PCR buffer, 15 pmol of each primer, 100 *μ*mol/L of each dNTP, and one unit Taq DNA polymerase (Cinnagen, Iran). PCR was carried out for 35 cycles of 45 s at 94°C, 60 s at 65°C (P55 primers), 66°C (IDS primers), and 58°C (G6PD primers), 60 s at 72°C and the final cycle at 72°C, for 5 mins.

 The G6PD gene contains a silent polymorphism (C/T) at nucleotide 1311. In the first round primers sense A and antisense D were used (Table 1). In the second round, 1 *μ*L of the first round PCR product was added to 49 *μ*L of fresh reaction mix and reamplified using 15 pmol each of primers sense F and antisense M. Primer M contains two mismatches to the gene sequence to generate a *Bcl1* site if the T-allele is present.

 The IDS gene was amplified using primers IDS-UD and IDS-DD. This gene contains a silent polymorphism (C/T) at nucleotide 438. The P55 gene was amplified using primers P55-UDA and P55-DDM. This gene contains a silent polymorphism (G/T) at nucleotide 458. To identify heterozygotes and amplify the DNA sequence bearing these polymorphisms a mismatched base was introduced into the antisense primers to create a *Bcl1 * and *Bsh1236 I* restriction site during the PCR if the C and G alleles were present, respectively.

### 2.5. RNA Extraction

 Total RNA was extracted from cell pellets using Tri-reagent (Sigma).

### 2.6. Reverse Transcription Polymerase Chain Reaction (RT-PCR)

#### 2.6.1. cDNA Synthesis

 For G6PD, total cellular RNA from granulocytes or lymphocytes was incubated at 70–80°C for 3 minutes to remove secondary structure. The reaction mix was made up to 20.2 *μ*L. Final volume containing 50 *μ*L antisense primer D, 1X first strand buffer, 50 *μ*M each dNTP, 25U RNasin (Promega), 250 units MMLV-RT (Gibco-BRL), incubated at 42°C for 1 h, and denatured at 70–80°C for 10 min. cDNA synthesis for IDS and P55 RNA was performed similarly, except that random primers (0.4 *μ*g/reaction, Promega) were used and the reaction was incubated at room temperature for 10 min before incubation at 42°C. 

### 2.7. Amplification

To amplify the G6PD cDNA, 20 *μ*L of RT reaction was made up to 100 *μ*L in 1X PCR buffer, 10 *μ*M of primer sense A and 1 unit Taq DNA polymerase. The amplification carried out for 40 cycles of 1 min at 94°C, 1 min at 58°C and 1 min at 72°C. The amplified products were re-amplified with mismatched primers F and M in the second round.

To amplify the IDS and P55 cDNAs, 4 *μ*L of RT reaction were used with primers IDS-SC and IDSA.

### 2.8. Digestion of PCR and RT-PCR Products

PCR products were digested either by *Bsh*1236 I or *Bcl*1 for P55, IDS, and G6PD polymorphisms, respectively. A reaction mix was made containing 1 *μ*L of appropriate 10X buffer, 1 *μ*L of enzyme (Fermentase) and 8 *μ*L of RT-PCR products, incubated at 37°C overnight for *Bsh*1236 I and 1 h at 55°C for *Bcl*1. Digested products were separated by electrophoresis on 4% Nu-Sieveagarose gel containing ethidium bromide and were visualized using an UVP Image store 7500 (UVP Life Science, UK). The image was saved on disk and the bands were quantitated using ImagerSoft ID2D software. 

### 2.9. Role of the Funding Source

This work was supported by funds from Iranian Molecular Medicine Network and Zanjan University of Medical Sciences. Funding source in this study had no involvement in study design, the collection, analysis or interpretation of data and writing of the paper.

## 3. Results

### 3.1. Genotyping for the G6PD Gene

**Figure 1:**
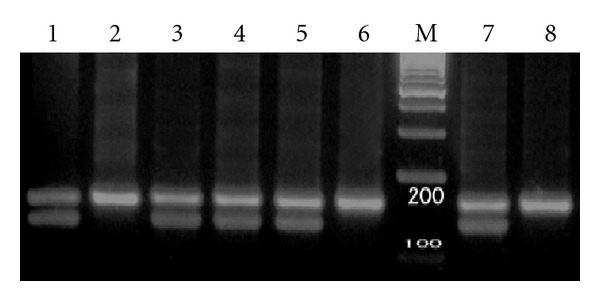
Genotyping by *Bc1I* digestion of PCR products. Lanes 1, 3, 4, 5, and 7 C/T; lanes 2 and 6 C/C; M, size marker 100 bp; lane 8, undigested PCR product.

 200 normal individuals and 45 patients with AML were first screened for heterozygosity at nt.1311 of the G6PD gene. DNA from either granulocytes or lymphocytes was amplified by PCR using nested primers spanning exons 10 and 11. Products were digested with *Bcl*1 and examined by electrophoresis on 4% NuSieve agarose gel. Heterozygotes showed two distinguishable bands of 207 and 184 bp corresponding to the C and T alleles, respectively (Figure 1).

 Fifty eight out of 200 normal individuals (29%) and 13 out of 45AML patients (28.9%) were found to be heterozygous. Our results showed that overall 29% females, in this Iranian population were heterozygous for this polymorphism.

### 3.2. Genotyping for the P55 Gene

**Figure 2:**
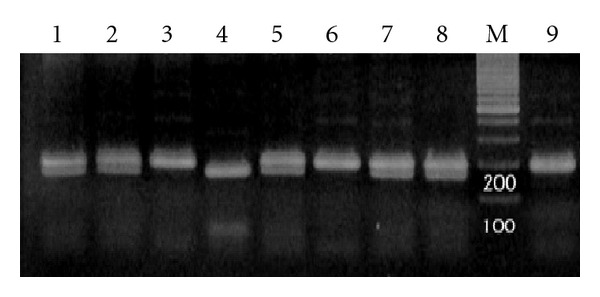
Genotyping by *Bsh1236 I* digestion of PCR products. Lanes 1, 2, 5, 7 and 8 G and T alleles (G/T); lanes 3 and 6, G/G; lane 4, T/T; M, size marker 100 bp; lane 9, undigested PCR product.

 All normal individuals and patients with AML were screened for heterozygosity at nt. 458 of P55 gene. Heterozygotes showed two distinguishable bands of 191 bp and 178 bp corresponding to the G and T alleles for P55 gene, respectively, (Figure 2). 

 Heterozygosity rates for these genes in our normal individuals were 97/200 (48/5%) and in AML patients were 48.5% (22 out of 45 patients). The overall 48.6% females were heterozygous for this polymorphism.

### 3.3. Genotyping for the IDS Gene

**Figure 3:**
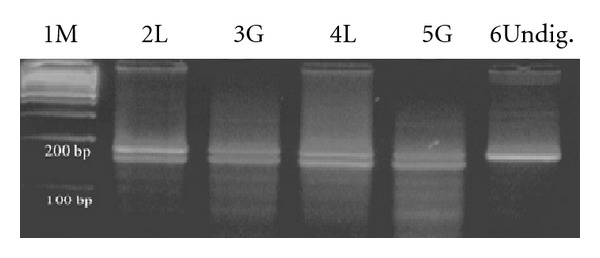
Clonality analysis by *Bsh1236 I* digestion of RT-PCR products of normal individuals lane1, 100 bp size marker; lanes 2–5, lymphocytes and granulocytes (Polyclonal); lane 6, undigested RT- PCR product.

All normal individuals and patients with AML were screened for heterozygosity at nt. 438 of IDS gene. Heterozygotes showed two distinguishable bands of 160 and 130 bp for IDS gene corresponding to the C and T alleles (Figure 3).

78 subjects (39%) out of 200 normal individuals and 19 patients (42.8%) out of 45AML patients were found to be heterozygous. Our results showed that, overall, 39.7% females were heterozygous for this polymorphism.

### 3.4. Clonality Analysis of Normal Females

One hundred normal individuals who were heterozygote for one of the G6PD, P55, or IDS genes were studied for clonality by RT-PCR. Any samples that showed the expression of more than 85% of one allele was classified as “clonal pattern.” 

**Figure 4:**
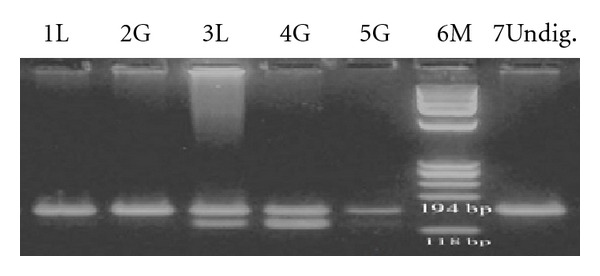
Clonality analysis by *BclI* digestion of RT- PCR products of normal females. Lanes 1, and 2, lymphocytes and granulocytes representing the 191 bp band only (monoclonal); lanes 3–5, lymphocytes and granulocytes representing both 191 bp and 178 bp bands (polyclonal); M, size marker *φ*X174; lane 7, undigested RT- PCR product.

Ninety two normal female individuals (92%) showed a polyclonal pattern and 8/100 individuals showed a clonal pattern (Figure 4).

### 3.5. Clonality Analysis of Female Patients with AML

**Figure 5:**
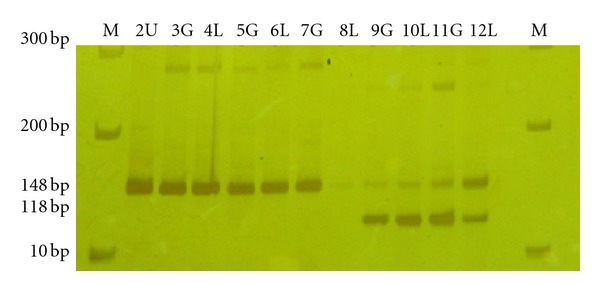
Clonality analysis by *BclI* digestion of RT- PCR products of AML patients at long followup during remission. Lanes 3–8 representing a clonal pattern (the 160 bp band only) in both G and L. Lanes 9–12 representing a polyclonal pattern (bands of 160 and 130 bp). M = 100 bp Marker; U = undigested 160 bp; G = granulocyte; L = lymphocyte.

At presentation, only one out of 45 females showed a polyclonal pattern and 44 out of 45 individuals showed a clonal pattern of X-chromosome inactivation in both lymphocytes and granulocytes. Twenty seven patients were available for a longer followup after remission. Twenty three out of 27 patients showed a clonal pattern in their B and T lymphocytes and granulocytes and 4 out of 27 patients showed a polyclonal pattern in all cell lineages during remission (Figure 5).

Ten out of 27 patients were available for a longer followup (up to 12 months) (Table 2). Seven patients showed a clonal pattern in all cell lineages and 3 patients showed a polyclonal pattern in their B and T lymphocytes and granulocytes. 

## 4. Discussion

 A neoplastic cell population is the progeny of single cell with a genetic insult, with the same X-chromosome in the active or inactive state. Therefore, if only one X-chromosome can be shown to be active in all the cells of tissue sample, this is evidence for clonality. Many primary studies on hematological disorders have given evidence for the clonal origin of most hematological malignancies and have suggested that these neoplasms arose from an acquired mutation in a single progenitor cell [15–18]. Therefore, clonality analysis can play an important role in the study of malignancies.

Within the last decades, methods using mRNA for demonstration of silent polymorphism of a single nucleotide in a small number of cells have been introduced as a sensitive assay for detection of monoclonality independent of the differential methylation analysis [19]. The aim of this study was to evaluate the clonality in 200 healthy individuals and 45 female patients with AML aged 20–45 years before the treatment and during remission at the RNA level.

### 4.1. The Prevalence of Heterozygosity

By using RNA polymorphisms, we have detected expression of both alleles in the large majority of heterozygous females. The initial screening for heterozygous status at the P55, G6PD, and IDS loci revealed that 114/245 (48.5%) of the females were heterozygous for P55, 48.5% for IDS, and 28.9% for G6PD gene. These rates are approximately similar to the reports of the other studies. Luhovy and colleagues showed 48% heterozygosity for P55 gene in their study population [20]. El Kassar and Hopwood reported 50% heterozygosity for IDS gene [19, 21]. We had obtained 43% heterozygosity for IDS and 23.4% for G6PD gene in our previous study on English population (Caucasian) [22]. In this study, in order to increase the rate of heterozygosity detection we used IDS, G6PD and P55 genes simultaneously.

### 4.2. X-Chromosome Inactivation Analysis in Healthy Control Women

Two hundred healthy women were assessed as controls. One hundred women were heterozygous for at least one of the three aforementioned gene polymorphism. 48.5% were heterozygous for P55, 39% for IDS, and 29% for G6PD gene. Among healthy controls we observed monoclonality of granulocytes and lymphocytes in 8 cases (8%) and 92% had a polyclonal pattern. 

El Kassar and colleagues in a study using two different techniques including DNA methylation pattern of human and rogen receptor gene and transcript analysis of the IDS, P55, and G6PD genes assessed X-chromosome inactivation pattern in PMN, platelet, and T lymphocytes of 48 patients with essential trombocytosis and 59 healthy women. Their study showed 9% clonality using DNA methylation analysis and 2.4% using transcript polymorphism analysis in healthy female controls [23]. 

In the our previous study, we also investigated the expression of polymorphisms at G6PD and IDS genes at the RNA level using nonradioactive RT-PCR method in 26 aplastic anemia patients and 35 normal females showed 3/35 (9%) clonallity pattern in normal controls [24]. However, by chance variation, in a small number of normal individuals the majority of cells will have inactivated one rather than the other X-chromosome, so called extreme lionisation. Extreme lionization was reported in 4–23% of normal population. 

Fialkow [25] in a study of 241 normal females heterozygous for G6PD isoenzymes observed only 2 females who showed extreme lionisation. Also Vogelstein et al. [26] reported unilateral Lionizationin 4% of normal females. There are more recent studies that have suggested higher rates of extreme lionization. Gale et al. [27] showed extreme lionization in 23% of normal females using PGK and HPRT probes and 22% by M27*β* probe [8]. It is notable that these studies used DNA methylation to define the active X, thus the observed rate of extreme lionization might be related to the methods used. Therefore in contrast Prchal et al. [28] reported no cases of pseudoclonality among 200 normal females studied by RT-PCR and ligase detection reaction for the G6PD nt.1311 polymorphism. 

 G6PD isoenzyme electrophoresis directly measures gene activity on the two X-chromosomes, whereas in the DNA methylation methods X inactivation is inferred indirectly. DNA methylation may not always correlate with X inactivation consistently. In some leukemic samples hypermethylation of both alleles has been reported while they were studied by southern blotting [8, 29]. Therefore the possibility of obtaining false positive results should be taken into consideration.

### 4.3. X-Chromosome Inactivation Analysis in AML Patients before Treatment

In our study, forty five de novo AML patients were heterozygous for IDS, P55, and G6PD genes and were assessed for X-chromosome inactivation. At presentation, nearly all the cells originated from mutant stem cell and clonality was observed in all cells. 44/45 (AML female patients) (97.8%) in our study showed a clonal pattern in their granulocytes. Patients number 34 and 36 showed a polyclonal pattern in their lymphocytes. One patient showed a polyclonal pattern in both granulocytes and lymphocytes. These data are predictable for presentation phase of AML as most of the blasts originating from the abnormal clone.

O'Malley et al. in a study of clonality evaluation of spleen in 17 patients with AML showed 40% clonal pattern [30].

### 4.4. X-Chromosome Inactivation Analysis in AML Patients at Remision Phase

Twenty seven patients out of 45 AML patients were available after treatment. 4/27 patients converted from clonality to polyclonality in granulocytes and lymphocyte lineages and 23/27 patients (85.2%) remained clonal at different time points during the remission phase. These data may suggest that chemotherapy may ablate the overtly leukemic clone, but spared some preleukemic clonal stem cells. However, the current available data do not suggest that clonal remission is associated with a rapid relapse, as our 4 patients are at remission with no relapse in one-year followup. Another possibility for clonal remission in AML is that repopulation of the marrow has happened by only one or a few normal surviving stem cells, as has been published in mice marrow transplantation [31]. Recently, Ding et al. have studied clonal remission using whole-genome sequencing of eight AML patients at relapse. They concluded that AML relapse is associated with the new mutations and clonal evolution patterns, which is caused by chemotherapy [32].

Fialkow and colleagues studied X-chromosome inactivation pattern of hematopoeisis in 27 patients with acute nonlymphocytic leukemia who were heterozygous for the X-chromosome-linked enzyme G6PD. They showed 6/27 (22.2%) of elderly patients and 16/27 (59.2%) of young patients developed a clonal pattern in erythrocytes and platelets. 3/27 (11.1%) of patients whose progenitor cells were involved by the leukemia exhibited clonal pattern of hematopoeisis. Out of 13 patients who followed at remission phase, 8 patients (61.5%) showed polyclonal pattern. This result is in contrast with our findings [15]. 

Gale and her colleagues assessed X-chromosome inactivation patterns with differential methylation patterns of heterozygotes for three DNA probes, HPRT, PGK, and M27*β*, in 77 female patients with AML and 75 normal controls. they reported that blast cells from 67 out of 68 analysable samples (99%) were monoclonal or had a skewed X-inactivation pattern. A skewed pattern in remission was also found in 26 of 77 patients (34%), but in 16/75 (21%) of normal controls [27]. 

Jinnai and colleagues [33] studied clonal analysis using X-chromosome inactivation patterns of the phosphoglycerate kinase (PGK) and DXS255 (M27 beta) genes in 34 women with AML at remission phase. 25/34 (73.6%) of patients showed polyclonal pattern in G and MN cells and 9/34 (26.4%) showed clonal hematopoeisis. Three out of 9 patients (30%) showed clonal pattern in MN cells and 6 patients had clonal pattern in both G and MN cells. When clonal patterns are different in patients with the same disease, it seems important to elucidate their clinical implications.

### 4.5. Clonality Pattern in AML Patients in a Long-Term Followup after Treatment

10/27 patients after the treatment were available for a longer followup. These patients were followed up to 12 months. 7/10 patients (70%) preserved clonal patterns and three patients converted to polyclonal patterns in G and L lineages. 

We expect that after chemotherapy and the induction of remission, hematopoeisis in AML patients convert from clonal to polyclonal, but this does not happen in all the patients and some patients remain clonal after remission. 

As mentioned before, many healthy females have clonal hematopoeisis at the normal condition. So, it can be concluded that each patient who showed clonal hematopoeisis at remission phase, may have had a clonal pattern before the disease or maintained their clonal pattern during remission. Since we did not have the patient samples before the start of the AML, so we could not come to a clear conclusion regarding clonality. Our results showed 8% (8/100) clonality in healthy controls compared with 85% (23/27 patients) at remission, which is statistically significant (*P* < 0.01). This significant difference suggests that the results of the clonality in most of the patients are true. 

The important question is whether the rate of relapsing is higher in patients with clonal hematopoeisis at remission time or not? A study showed that after transplantation of a small number of hematopoetic stem cells to the mice models, efficient clonal hematopoeisis was developed by few stem cells through animal's life time and survived them [31]. Another study showed that hematopoeisis in radioexposed cats that were transplanted with hematopoetic stem cells remained clonal after many years without any complications [34]. These data could suggest that the clonality in remission phase does not necessarily mean relapse of the disease. In contrast, some studies did not support this theory [32].

To answer this question longer followup of more patients at the remission phase is nessecary. 

Overall, we showed 97.8% clonality in AML patients at presentation, 85.2% immediately after remission, and 70% at a long-term followup after chemotherapy.

From this study, it can be concluded that clonality at remission is a frequent event in AML and does not necessarily mean relapse of the disease. There is also a possibility of the conversion of clonality to polyclonality over time after chemotherapy. However, larger number of patients with a longer followup is necessary to answer this question.

## Figures and Tables

**Table 1:** PCR primer sequences and the restriction enzymes.

Designation	Description	Sequence	Restriction enzyme
IDS-SD	IDS sense DNA primer	5′-GCCCCAAAGAAGGGAGGGTCC-3′	*Bcl1*
IDS-SC	IDS sense cDNA primer	5′-TGTACGACTTCAACTCCTACTGGA-3′	
IDS-A	IDS antisense DNA primer	5′-TGGAAAAGACCAGCTATACGGAGAATGAT-3′	

P55-UDA	P55 sense DNA primer	5′-CTCCTCAAAGCAGGCTTCATGCCTG-3′	*Bsh*1236I
P55-DDM	P55 antisense DNA primer	5′-CGTACAGGACTGTTTTTCATTCAGCTTCCG-3′	
P55-UR	P55 sense cDNA primer	5′-CACAGAAGAGCCCATGGGAATCGC-3′	
P55-DR	P55 antisense cDNA primer	5′-CGCCTTCTGCAGCTGATCCAC-3′	

G6PD-A	G6PD Outer sense primer	5′-GACCAAGAAGCCGGGCATGTT-3′	*Bcl1*
G6PD-D	G6PD Outer antisense primer	5′-GGTGCAGTGGGGTGAAA-3′	
G6PD-SF	G6PD inner sense primer	5′-TGTTCTTCAACCCCGAGGAGT-3′	
G6PD-SM	G6PD inner antisense primer	5′-AAGACGTCCAGGATGAGGTGATC-3′	

**Table 2:** Features of the patients who were followed after remission.

Patients number	Age	Diagnosis (FAB)	Clonality	Duration of following (month)	Cytogenetics	Flow cytometry
26	40	M_3_	Mono.	8	46XX, t(15; 17) (q22; q21)[2]/46, XX[5]	Compatible with M_3_
28	20	M_3_	Mono.	12	46XX, t(15; 17) (q22; q21)[3]/46, XX[11]	Compatible with M_3_
33	21	M_3_	Mono.	6	46XX, t(15; 17) (q22; q21)[5]/46, XX[15]	Compatible with M_3_
35	43	M_3_	Mono.	6	46XX, t(15; 17) (q22; q21)[14]/46, XX[19]	Compatible with M_3_
36	45	M_3_	Mono.	6	46XX, t(15; 17) (q22; q21)[7]/46, XX[13]	Compatible with M_3_
40	21	M_3_	Poly.	7	46XX, t(15; 17) (q22; q21)[5]	Compatible with M_3_
43	20	M_2_	Poly.	5	46XX, t(15; 17) (q22; q21)[3]/46, XX[17]	Compatible with M_2_
45	34	M_2_	Mono.	4	N.A	N.A
47	40	M_3_	Mono.	6	46XX, t(8; 15; 17) (q24.10; q21)ort(8; 14)(q11; q32), t(15; 17) (q22; q21)[6]/46, XX[12]	Compatible with M_3_
48	20	M_3_	Poly.	5	46XX, t(15; 17) (q22; q11–21)[12]/46, XX[8]	Compatible with M_3_
